# Determinants of emergency response willingness in the local public health workforce by jurisdictional and scenario patterns: a cross-sectional survey

**DOI:** 10.1186/1471-2458-12-164

**Published:** 2012-03-07

**Authors:** Daniel J Barnett, Carol B Thompson, Nicole A Errett, Natalie L Semon, Marilyn K Anderson, Justin L Ferrell, Jennifer M Freiheit, Robert Hudson, Michelle M Koch, Mary McKee, Alvaro Mejia-Echeverry, James Spitzer, Ran D Balicer, Jonathan M Links

**Affiliations:** 1 Johns Hopkins Preparedness and Emergency Response Research Center, 615 North Wolfe Street, Room E7537, Baltimore, MD 21205, USA; 2 Department of Environmental Health Sciences, Johns Hopkins Bloomberg School of Public Health, 615 North Wolfe Street, Baltimore, MD 21205, USA; 3 Johns Hopkins Public Health Preparedness Programs, 615 North Wolfe Street, Room E7537, Baltimore, MD 21205, USA; 4 Department of Biostatistics, Johns Hopkins Bloomberg School of Public Health, 615 North Wolfe Street, Baltimore, MD 21205, USA; 5 Department of Health Policy and Management, Johns Hopkins Bloomberg School of Public Health, 615 North Wolfe Street, Baltimore, MD 21205, USA; 6 Eastern Idaho Public Health District, 1250 Hollipark Drive, Falls, ID 83401, USA; 7 Virginia Department of Health-Lord Fairfax Health District, 10 Baker Street, Winchester, VA 22601, USA; 8 University of Wisconsin-Milwaukee, 1828 East Rusk Avenue, Milwaukee, WI 53207, USA; 9 Butler County Health Department, 1619 North Main Street, Poplar Bluff, MO 63901, USA; 10 Meeker County Public Health, 114 N Holcombe Avenue, Suite 250, Litchfield, MN 55355, USA; 11 Marion County Health Department, 3838 North Rural Street, Indianapolis, IN 46205, USA; 12 Miami-Dade County Health Department, 8600 NW 17th Street, Suite 200, Doral, FL 33126, USA; 13 Multnomah County Health Department, 426 SW Stark Street, 7th Floor, Portland, OR 97224, USA; 14 Department of Epidemiology, Faculty of Health Sciences, Ben-Gurion University of the Negev, P.O.B. 653, Beer-Sheva 84105, Israel; 15 Clalit Research Institute, Clalit Health Services, 101 Arlozorov Street, Tel-Aviv, Israel

## Abstract

**Background:**

The all-hazards willingness to respond (WTR) of local public health personnel is critical to emergency preparedness. This study applied a threat-and efficacy-centered framework to characterize these workers' scenario and jurisdictional response willingness patterns toward a range of naturally-occurring and terrorism-related emergency scenarios.

**Methods:**

Eight geographically diverse local health department (LHD) clusters (four urban and four rural) across the U.S. were recruited and administered an online survey about response willingness and related attitudes/beliefs toward four different public health emergency scenarios between April 2009 and June 2010 (66% response rate). Responses were dichotomized and analyzed using generalized linear multilevel mixed model analyses that also account for within-cluster and within-LHD correlations.

**Results:**

Comparisons of rural to urban LHD workers showed statistically significant odds ratios (ORs) for WTR context across scenarios ranging from 1.5 to 2.4. When employees over 40 years old were compared to their younger counterparts, the ORs of WTR ranged from 1.27 to 1.58, and when females were compared to males, the ORs of WTR ranged from 0.57 to 0.61. Across the eight clusters, the percentage of workers indicating they would be unwilling to respond regardless of severity ranged from 14-28% for a weather event; 9-27% for pandemic influenza; 30-56% for a radiological 'dirty' bomb event; and 22-48% for an inhalational anthrax bioterrorism event. Efficacy was consistently identified as an important independent predictor of WTR.

**Conclusions:**

Response willingness deficits in the local public health workforce pose a threat to all-hazards response capacity and health security. Local public health agencies and their stakeholders may incorporate key findings, including identified scenario-based willingness gaps and the importance of efficacy, as targets of preparedness curriculum development efforts and policies for enhancing response willingness. Reasons for an increased willingness in rural cohorts compared to urban cohorts should be further investigated in order to understand and develop methods for improving their overall response.

## Background

Effective response to public health emergencies is based not only on the ability of the members of the public health workforce to respond, but also on their willingness to respond (WTR). Previous research has identified ability as conceptually and operationally distinct from willingness [1,2] and has suggested that WTR is rooted in risk perception theory [3]. Studies have pointed to WTR as a function of the interplay between perceptions of threat and efficacy toward a hazard [4,5]. Research has also identified WTR as a requisite for quality in public health systems' response efforts, as response willingness is fundamental to response capacity [6]. Examples in which low WTR had the potential to hinder public health and healthcare response to an infectious disease outbreak have been reported [4,7-11].

The above insights are salient in light of the Institute of Medicine's recognition of limits in emergency response surge capacity [12]. Such limitations are germane to local health departments (LHDs), which are at the hub of the public health emergency preparedness system [13] but have faced significant staffing constraints due to fiscal austerity. A survey of U.S. LHDs in early 2010 revealed that between January 2008 and December 2009, these agencies collectively lost approximately 15% of their workforce due to budget cutbacks, and nearly half of all LHDs anticipated further budget cuts [14].

Given the need for LHDs to meet all-hazards response expectations despite staffing constraints, the local public health workforce's emergency response willingness has profound implications for health security in the face of an ever-broadening array of threats. Previous research among non-public health agency responders has found WTR to be scenario-specific [2]. Thus far, research on LHD workers' WTR has focused on pandemic influenza as the scenario of interest [10,11], leaving important gaps in understanding this occupational cohort's relative attitudes toward response to other categories of public health threats. Further, prior LHD-based WTR research has not explicitly addressed potential jurisdictional differences in WTR by urban versus rural categorization. Such a comparative examination is relevant because research has pointed to distinct challenges following a public health emergency based on jurisdictional size and related infrastructure considerations [15]. Moreover, research to date on WTR among LHD workers has been relatively circumscribed in its jurisdictional coverage [10,11].

A more robust, scenario-based understanding of the local public health workforce's response willingness is critical for gauging public health system preparedness within the all-hazards spectrum and for identifying potential avenues to enhance it. In an effort to address some of these gaps, the present study's aims are to assess determinants of local public health workers' response willingness toward a range of representative public health emergency scenarios, in a set of geographically diverse urban and rural U.S. public health jurisdictions.

## Methods

### Study design and implementation

Research ethics approval for the study was received from the Johns Hopkins Bloomberg School of Public Health Institutional Review Board (JHSPH IRB) (exempt status # 45 CFR 46.101 (b) (2)). Per JHSPH IRB approval, written consent from the participants was not required, because the research presented no more than minimal risk to subjects and involved no procedures for which written consent is normally required. The JHSPH IRB-approved materials included a written disclosure describing the study, and emphasizing voluntary participation. Verbal consent was not requested or required by JHSPH IRB for this approved study.

The research team provided an online survey, the Johns Hopkins ~ Public Health Infrastructure Response Survey Tool [JH ~ PHIRST], to assess emergency response willingness among public health workers in eight LHD clusters. Each cluster included three or more proximate or contiguous counties/jurisdictions. Based on U.S. Census data [16] and consistent with previous population-related research on LHDs [17], a rural cluster was defined as one where the average LHD serves residents in county(ies) whose average population is under 50,000 residents, whereas an urban cluster was defined as one where the average LHD serves residents in county(ies) whose average population is greater than 50,000 residents.

Convenience sampling was used for cluster recruitment, based on previously identified professional contacts from each cluster, with an effort toward achieving geographic and jurisdictional diversity. Primary contacts were asked to identify and invite neighboring counties/jurisdictions to consider participation in the project via a snowball sampling approach, to reach a three-county per cluster minimum. In most cases, the primary contacts made efforts to engage and identify more than three counties per cluster. A Collaboration Agreement was signed by each participating LHD within a cluster. After all administrative forms were processed and participation was confirmed, researchers worked directly with cluster contacts to set a mutually agreed-upon date to launch the baseline survey. On the date of the baseline survey launch, LHD contacts were sent an email that they forwarded to their employee listserv announcing the start of the baseline survey. In the email, employees were asked to click on an embedded link that took them to a registration site independent of the data collection/analysis process. The registration site required them to create an account and set up a username and password, which then generated a personalized link containing a unique, randomly-created identifier to SurveyMonkey (SurveyMonkey.com, Portland, OR) for completion of the baseline survey. This unique identifier helped mitigate duplication of surveys in instances where an employee only partially completed the survey in a session, and provided user-anonymity during the survey and subsequent data analysis

The voluntary survey took approximately 15-20 minutes to complete and was scheduled to remain open for a period of four weeks. Weekly updates were provided to each cluster's primary contacts via email which included LHD response rates for their health departments. Reminder emails could then be sent out through their listservs, without indication of who had responded or not. The aim was to achieve an 80% response rate from each LHD during the survey period, and extensions to the four-week window were provided on request.

### Survey contents and analysis

The *JH ~ PHIRST *is an anonymous online survey instrument consisting of a demographic section and attitude/belief sections focusing on health department workers' attitudes and beliefs toward public health emergency response, for each of four emergency scenarios (weather-related event, pandemic influenza, radiological 'dirty' bomb terrorism event, and inhalational anthrax bioterrorism event). For each scenario, respondents were also asked for their willingness to respond in three contexts: if required, if asked but not required, and regardless of severity. Responses to the attitude/belief statements were based on a 9-point Likert scale with a response of '1' indicating strong agreement with the question, a response of '5' indicating neutrality, and a response of '9' indicating strong disagreement with the question. Respondents could also indicate "don't know."

Prior to analysis, responses to the attitude and belief statements were dichotomized into categories of ≤ 4 ('positive response') versus > 5 ('negative response'). This dichotomized approach was applied in accordance with similar previous studies performed using close variations of this survey tool [4,5]. Levels of WTR were evaluated from several perspectives: clusters, urban/rural designation, respondent demographic characteristics and respondent attitudes/beliefs regarding public health emergency response.

Witte's Extended Parallel Process Model (EPPM) is useful for understanding determinants of WTR to public health emergencies [5,10]. The EPPM predicts that, in the face of uncertain risk, people are more apt to engage in proactive behaviors if they perceive the threat associated with a hazard as legitimate and can address a desired intervention efficaciously. One of the four EPPM categories was assigned to each respondent using the threat and efficacy dimensions (each comprised of two relevant attitude/belief statements - Methodology1, Additional file 1), with low and high perceived threat and efficacy categories calculated as described in previous EPPM survey-based research [11]. Summary statistics were calculated for demographic characteristics and for the WTR contexts by cluster and urban/rural designation. Generalized linear latent and mixed models (GLLAMM) analyses [18] were performed to compare the dichotomized WTR responses between demographic characteristics, urban/rural designation, EPPM categories, and attitude/belief statements. These multilevel analyses were adjusted for all demographic characteristics in the survey, and adjusted for potential correlations between attitudes/beliefs among participants within an LHD and among LHDs within a cluster. Responses to the attitude/belief statements were compared between the urban/rural designations and across EPPM categories. The Wald test [19] was calculated as an overall assessment of differences in predictors with more than two categories (e.g., job classification). *P*-values of 0.05 were considered statistically significant for these analyses; results with *p*-values > 0.05 were noted as "NS" for non-significant. All analyses were performed using STATA version 11.1 (STATA Corp., College Station, TX, 2010).

## Results

A total of eight LHD clusters (out of eight recruited clusters, from nine U.S. states), were given access to the survey and responded to it starting in April 2009 and ending in June 2010 (Table S1 [Supplementary tables, designated by "S", are unpublished, but are available for review by contacting the corresponding author]). Four of these eight clusters were considered rural (in Idaho, Minnesota, Missouri, and Virginia) and four were considered urban (in Florida, Indiana, Oregon/Washington, and Wisconsin). Respondents from the rural clusters ranged in number from 75 to 216 for a total of 630; respondents from the urban clusters ranged in number from 138 to 1184 for a total of 2363. The overall response rate was 66% based on numbers of eligible respondents provided by the LHDs. Table 1 shows the distributions of respondents' demographic characteristics by urban/rural designation and over all respondents. The largest difference between urban and rural demographics was in education with 58% of rural and 67% of urban respondents having at least a bachelor's degree. The next largest difference occurred for gender with 88% and 81% female respondents in the rural and urban clusters, respectively. Rural clusters had 62% and urban clusters 57% of their respondents with five or more years in the organization. Table S2 shows the distribution of the demographic characteristics by the individual clusters.

**Table 1:** Respondent characteristics by urban/rural designation

	Designation^a^		
**Respondent Characteristics^b^**	**Rural**	**Urban**	**Overall**

Females(%)	87.5	81.2	82.6

Age: 40 years +(%)	70.2	69.8	69.9

Bachelors Degree +(%)	57.8	67.2	65.3

Work in organization 5 years +(%)	62.1	56.7	57.9

Work in profession 10 years +(%)	52.9	48.7	49.6

First responder role(%)	35.9	35.8	35.8

Dependent family member(%)	67.4	65.7	66.1

Number of respondents	630	2363	2993

^a ^Rural clusters include: MO, MN, ID, VA; urban clusters include: FL, WI, OR/WA, IN

^b ^Characteristics are binary, e.g. Female and Male, Work in organization < 5 years and 5 years+

**Figure 1:**
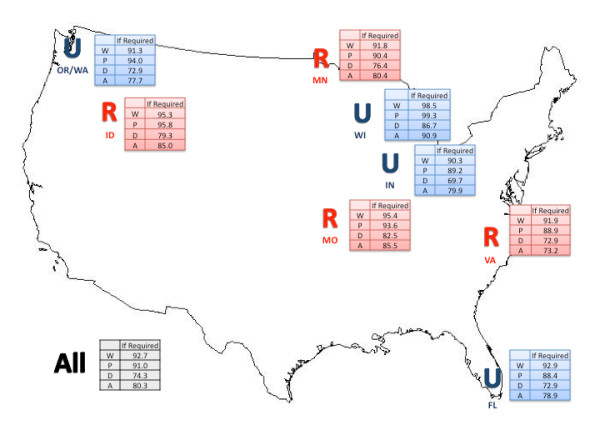
**Percent agreement for willingness-to-respond if required across clusters and disaster scenarios**. Cluster (State): FL(Florida), MO(Missouri), MN(Minnesota), ID(Idaho), VA(Virginia), OR/WA(Oregon/Washington), WI(Wisconsin), IN(Indiana). Classification: U- Urban, R-Rural. Disaster Scenario: W - Weather-related, P - Pandemic Influenza, D - Radiological 'dirty' bomb, A - Anthrax bioterrorism.

Figure 1 shows the levels of WTR for the if required context, across scenarios, by cluster and indicating the urban/rural classification of each cluster. Table 2 shows the levels of WTR by the 12 context/scenario combinations for the urban and rural respondents, and all respondents. Rural respondents had higher levels of WTR than urban respondents with the differences across these WTR context/scenario combinations ranging from 2 to 12% (Table 2). The largest of these differences (8 to 12%) for the if asked and regardless of severity WTR contexts, occurred for the pandemic influenza, radiological 'dirty' bomb, and anthrax bioterrorism scenarios. Over all respondents, WTR levels in the radiological 'dirty' bomb and anthrax bioterrorism scenarios were lower than for comparable contexts in the weather-related and pandemic influenza scenarios. For the if asked context, the overall WTR levels were 83% (weather-related scenario), 80% (pandemic influenza scenario), 69% (anthrax bioterrorism scenario), and 62% (radiological 'dirty' bomb scenario). The lowest overall WTR levels occurred for the regardless of severity context with 53% for the radiological 'dirty' bomb scenario. Comparing WTR contexts, the if required context generally had an 11% higher WTR level than if asked, and a range of 12% (pandemic influenza scenario) to 21% (radiological 'dirty' bomb scenario) higher WTR level than regardless of severity. Table S3 shows the levels of WTR by cluster for these 12 combinations.

**Table 2:** Percent agreement for willingness-to-respond (WTR) contexts by urban/rural designation of clusters and by scenario

		Designation^a^		
**Scenario**	**WTR context**	**Rural**	**Urban**	**Overall**

Weather- related	If required	94.0	92.3	92.7
	
	If asked but not required	86.8	81.7	82.8
	
	Regardless of severity	81.3	75.2	76.5

Pandemic influenza	If required	93.0	90.4	91.0
	
	If asked but not required	87.2	77.8	79.9
	
	Regardless of severity	86.5	76.3	78.6

Radiological 'dirty' bomb	If required	78.7	73.0	74.3
	
	If asked but not required	71.2	59.6	62.2
	
	Regardless of severity	59.3	51.3	53.1

Anthrax bioterrorism	If required	82.6	79.6	80.3
	
	If asked but not required	75.6	67.0	68.9
	
	Regardless of severity	70.4	62.9	64.6

^a ^Rural clusters include: MO, MN, ID, VA; urban clusters include: FL, WI, OR/WA, IN

Table 3 compares levels of WTR between the rural and urban respondents for the 12 context/scenario combinations with odds ratios (ORs) to assess the degree of association between the respondent groups and WTR. The analyses were adjusted for all demographic characteristics included in the survey, and take into account the within-cluster and within-LHD correlations of responses. The intraclass correlations for clusters and LHDs were low with all but a few instances ranging from < 0.001 to 0.10. For four of the 12 WTR context/scenario combinations, the OR and 95% confidence intervals (95%CI) showed significant differences between urban and rural respondents, with rural respondents having higher odds of self-reported response willingness than urban respondents (OR (95%CI): WTR regardless of severity for the weather-related scenario: 1.59 (1.24, 2.03); WTR if asked for the pandemic influenza scenario: 2.37 (1.72, 3.27); WTR regardless of severity for the pandemic influenza scenario: 2.24 (1.11, 4.54); and WTR if required for the radiological 'dirty' bomb scenario: 1.5 (1.13, 1.99).

**Table 3:** Comparison of willingness to respond between urban and rural designations of the clusters by scenario

	Emergency Scenarios			
	**Weather-related**	**Pandemic influenza**	**Radiological 'dirty' bomb**	**Anthrax bioterrorism**

	**Rural/Urban^a^**	**Rural/Urban**	**Rural/Urban**	**Rural/Urban**

**Willingness-to-respond context**	**OR(95%CI)^b^**	**OR(95%CI)**	**OR(95%CI)**	**OR(95%CI)**

If required	NS^c^	NS	1.5	NS

			(1.13, 1.99)	

If asked but not required	NS	2.37	NS	NS

		(1.72, 3.27)		

Regardless of severity	1.59	2.24	NS	NS

	(1.24, 2.03)	(1.11, 4.54)		

^a ^Rural clusters include: MO, MN, ID, VA; urban clusters include: FL, WI, OR/WA, IN

^b ^The reference category for the odds ratio and 95% confidence interval [OR(95%CI)] is the urban designation

^c ^NS - Not statistically significant at *p *= 0.05 level

When comparing categories within each demographic characteristic, adjusted for all the other characteristics, gender, age, education, first-responder role and having dependent family members showed consistent significant patterns of differences in WTR levels (Table S4). In six of the 12 WTR context/scenario combinations, the ORs that were statistically significant for WTR, comparing female to male respondents, ranged from 0.57 to 0.66. In eight of the 12 combinations, the significant ORs, comparing respondents at least 40 years of age to the younger respondents, ranged from 1.26 to 1.58. In seven of the 12 combinations, comparisons of WTR between respondents with at least a bachelor's degree and those with less education showed significant ORs from 1.26 to 1.90. Significant differences in WTR levels occurred in all context/scenario combinations for the first responder and dependent family member comparisons. The ORs, comparing respondents who considered themselves to be in a first responder role to those who did not consider themselves to be in that role, ranged from 1.7 to 2.6. The ORs for WTR, comparing respondents with a family member to care to those without that responsibility, ranged from 0.59 to 0.78.

Using the threat-efficacy perspective of the EPPM, comparisons of WTR levels across EPPM categories showed consistent patterns across almost all context/scenario combinations (Table 4). Respondents in the high-threat/low-efficacy (HT/LE), low-threat/high-efficacy (LT/HE), and high-threat/high-efficacy(HT/HE) categories had significantly higher odds of WTR than respondents in the low-threat/low-efficacy (LT/LE) category, where the smallest of these differences occurred for the HT/LE category. The largest differences in WTR, comparing the efficacy levels under the LT level (LT/HE to LT/LE categories), occurred in the if required context of the pandemic influenza scenario and anthrax bioterrorism scenario [OR(95%CI): 25.1(7.9, 79.6), 14.5(8.0, 26.1), respectively]. Large differences in WTR were also observed when comparing categories for the combined high vs combined low levels (HT/HE to LT/LE) for the if required context of the anthrax bioterrorism scenario, pandemic influenza scenario, and radiological 'dirty' bomb scenario [OR(95%CI): 19.6(11.5, 33.5), 18.9(8.2, 43.2), 14.8(9.7, 22.5), respectively]. For the LE level, respondents in the HT category had slightly higher odds of being willing to respond than those in the LT category (data not shown). For either level of threat, respondents in the HE category had considerably higher odds of being willing to respond than those in the LE category. For these efficacy comparisons, the range of ORs across WTR context/scenario combinations was wider for the LT category (4.6 to 25.1) than for the HT category (4.4 to 10.7).

**Table 4:** Comparison of willingness to respond by Extended Parallel Process Model (EPPM) categorization of cluster respondents by scenario

	Naturally-occurring Emergency Scenarios							
	**Weather-related**				**Pandemic influenza**			

	**EPPM Categories^a^**				**EPPM Categories**			

	**LT/HE**	**HT/LE**	**HT/HE**		**LT/HE**	**HT/LE**	**HT/HE**	

**Willingness-to-respond context**	**OR(95%CI)^b^**	**OR(95%CI)**	**OR(95%CI)**	**Wald p**	**OR(95%CI)**	**OR(95%CI)**	**OR(95%CI)**	**Wald p**

If required	10.29	2.32	10.3	< 0.001	25.09	3.02	18.82	< 0.001

	(4.72, 22.45)	(1.51, 3.56)	(5.12, 20.72)		(7.90, 79.64)	(1.97, 4.62)	(8.19, 43.23)	

If asked but not required	4.57	1.39	3.59	< 0.001	6.24	1.78	6.78	< 0.001

	(3.06, 6.82)	(1.05, 1.85)	(2.56, 5.02)		(4.06, 9.59)	(1.34, 2.37)	(4.64, 9.92)	

Regardless of severity	5.93	NS^d^	6.55	< 0.001	5.58	1.83	10.72	< 0.001

	(4.12, 8.54)		(4.68, 9.16)		(3.74, 8.32)	(1.39, 2.42)	(7.04, 16.34)	

	Terrorism-related Emergency Scenarios							

	Radiological 'dirty' bomb				Anthrax bioterrorism			

	EPPM Categories				EPPM Categories			

	LT/HE	HT/LE	HT/HE		LT/HE	HT/LE	HT/HE	

Willingness-to-respond context	OR(95%CI)	OR(95%CI)	OR(95%CI)	Wald p	OR(95%CI)	OR(95%CI)	OR(95%CI)	Wald p

If required	8.76	1.97	14.79	< 0.001	14.5	2.23	19.63	< 0.001

	(5.73, 13.39)	(1.45, 2.67)	(9.72, 22.49)		(8.04, 26.13)	(1.60, 3.09)	(11.51, 33.47)	

If asked but not required	7.25	1.47	8.18	< 0.001	8.24	1.88	11.01	< 0.001

	(5.14, 10.24)	(1.10, 1.95)	(6.04, 11.08)		(5.60, 12.13)	(1.40, 2.52)	(7.79, 15.54)	

Regardless of severity	9.85	1.21	12.94	< 0.001	8.78	1.57	13.18	< 0.001

	(7.02, 13.81)	(0.89, 1.65)	(9.48, 17.64)		(6.06, 12.72)	(1.17, 2.10)	(9.33, 18.60)	

^a ^EPPM categories: LT/LE - Low threat/low efficacy, LT/HE - Low threat/high efficacy, HT/LE - High threat/low efficacy, HT/HE - High threat/high efficacy

^b ^Reference category for odds ratio and 95% confidence intervals [OR(95%CI)] is LT/LE

^c ^Tests null hypothesis of no difference between EPPM categories

^d ^NS - Not statistically significant at *p *= 0.05 level

Difference in agreement with most attitude/belief statements comparing rural to urban respondents was most evident for the pandemic influenza scenario, with ORs of 1.5 to 2.9 for agreement regarding perceptions about severity of health consequences, likelihood of being asked to report, knowledge of public health impact, awareness and skills for responsibilities, ability to safely get to work, self-efficacy, and importance of one's role in agency response (Table 5). For the anthrax bioterrorism scenario, the ORs ranged from 1.4 to 2 for agreement regarding perceived likelihood of being asked to report, family prepared to function in absence, importance of one's role in agency response, and response efficacy.

**Table 5:** Comparison of agreement on attitude/belief statements between urban and rural designation of clusters by scenario

	Emergency Scenarios			
	**Weather-related**	**Pandemic influenza**	**Radiological 'dirty' bomb**	**Anthrax bioterrorism**

**Attitude/Belief Statements**	**Rural/Urban^a^**	**Rural/Urban**	**Rural/Urban**	**Rural/Urban**

	**OR(95%CI)^b^**	**OR(95%CI)**	**OR(95%CI)**	**OR(95%CI)**

Perceived likelihood of occurrence in this region	NS^c^	NS	0.7	0.6

			(0.51, 0.95)	(0.41, 0.88)

Perceived severe health consequences likely	NS	1.47	NS	NS

		(1.05, 2.05)		

Perceived likelihood of being asked to report to duty	NS	2.87	1.68	2

		(1.42, 5.80)	(1.17, 2.40)	(1.39, 2.90)

Perceived knowledge about the public health impact	NS	1.63	NS	NS

		(1.07, 2.47)		

Perceived awareness of role-specific responsibilities	2.24	1.71	NS	NS

	(1.74, 2.87)	(1.03, 2.83)		

Perceived skills for role-specific responsibilities	NS	2.12	NS	NS

		(1.29, 3.49)		

Psychologically prepared	NS	NS	NS	NS

Perceived ability to safely get to work	NS	1.9	1.45	NS

		(1.26, 2.86)	(1.10, 1.91)	

Confidence in personal safety at work	NS	NS	NS	NS

Perceived ability to perform duties (Self-Efficacy)	NS	2.02	NS	NS

		(1.36, 3.02)		

Perceived that family is prepared to function in absence	1.33	NS	1.42	1.36

	(1.02, 1.73)		(1.15, 1.75)	(1.04, 1.76)

Health Department's perceived ability to provide timely information	NS	NS	NS	NS

Perceived ability to address public questions	NS	NS	NS	NS

Perceived importance of one's role in the agency's overall response	NS	1.76	1.41	1.57

		(1.26, 2.45)	(1.00, 1.97)	(1.09, 2.26)

Perceived need for pre-event preparation and training	NS	NS	NS	NS

Perceived need for psychological support during event	NS	NS	NS	NS

Perceived need for post-event psychological support	NS	NS	NS	NS

Perceived high impact of one's response (Response Efficacy)	NS	NS	NS	1.37

				(1.01, 1.87)

^a ^Rural clusters include: MO, MN, ID, VA; urban clusters include: FL, WI, OR/WA, IN

^b ^The reference category for the odds ratio and 95% confidence interval [OR(95%CI)] is the urban designation

^c ^NS - Not statistically significant at *p *= 0.05 level

To evaluate the relationships between scenario-specific WTR and attitudes/beliefs regarding public health emergency response, these ORs (Table S5) were ranked from high to low within each WTR context/scenario combination. The following attitude/belief statements consistently ranked among the leading modifiers of WTR, revealing strong associations across the 12 context/scenario combinations: being psychologically prepared; perceived ability to safely get to work; confidence in personal safety at work; and perceived ability to perform duties (self-efficacy). Generally, the ORs for these attitude/belief statements were highest (17 to 28) for the pandemic influenza and anthrax bioterrorism scenarios under the WTR if required context. Under the WTR if required context, the perceived likelihood of being asked to report to duty, and under WTR regardless of severity, the perceived high impact of one's response (response efficacy), showed strong associations across all four scenarios.

## Discussion

A local public health workforce's willingness to respond (WTR) is an essential component of effective management of public health emergencies. Previous research on local public health agency workers has examined WTR in a pandemic influenza scenario [10,11], making this a novel study in its examination of WTR to multiple emergency scenarios among this occupational cohort and its identification of approaches that could be taken to improve their WTR. Although the National Response Framework encourages preparation for emergencies using an all-hazards approach [20], our results underscore that LHD workers' WTR is scenario-specific. WTR was shown to be greater for naturally-occurring emergency scenarios (weather-related and pandemic influenza) than for terrorism-related emergency scenarios (radiological 'dirty' bomb and anthrax bioterrorism) in all contexts (Table S3). Between the terrorism-related scenarios, workers' surveyed WTR was considerably lower for the radiological 'dirty' bomb.

These results suggest that all-hazards preparedness efforts may not be equally impactful on WTR among different hazards. Implementing effective scenario-based risk awareness campaigns among the LHD worker population may have an impact on increasing their knowledge, and thus their WTR [3]. Although warranting further study, these findings might also reflect the influences on WTR of scenario-based federal public health funding and related preparedness requisites for LHDs. In the U.S., such scenario-based funding and activities in LHDs have historically focused on bioterrorism (e.g. smallpox and anthrax dispersal) and pandemic influenza threats, which had higher WTR rates than for the radiological 'dirty' bomb scenario in our study.

Despite the geographic variability observed in WTR rates in this study, our findings point to gaps in response willingness as a significant surge capacity concern for public health response across a diverse array of threats. When employees across the eight LHD clusters were surveyed as to their WTR regardless of severity, the percentage of workers indicating they would *not *be willing to respond ranged from 14 to 28% for a weather-related event; 9 to 27% for pandemic influenza; 30 to 56% for a radiological 'dirty' bomb; and 22 to 48% for an inhalational anthrax bioterrorism event (Table S3). Given that severe public health threats require all employees at all skill levels, and in light of LHDs' central position within the public health emergency preparedness system, even the lower bounds of these ranges present a threat to this system's operational effectiveness. Moreover, previous research has shown local public health workers to be the most ready and willing among several civil service and hospital professional cohorts to respond to a pandemic influenza event [21]. In light of this previous research, the suboptimal levels of WTR observed across multiple scenarios among the current study's LHD cohort potentially point to even larger willingness gaps in a variety of responder cohorts, such as law enforcement and fire services. Indeed, multi-scenario-based gaps in WTR among police and fire departments merit further research examination.

We believe that this study is also the first to explicitly compare WTR rates between urban and rural local public health agencies. It is important to include rural LHDs in our investigation since only 4% of U.S. health departments serve populations greater than 500,000 [22] and approximately 20% of Americans live in rural areas [23]. Rural jurisdictions are not immune to the broad spectrum of potential public health threats. While urban areas may represent higher profile terrorism targets, rural jurisdictions could be affected by evacuation orders, decontamination considerations, or infectious disease spread. Since many rural areas house critical infrastructure, including elements of the food system and important bridges/roadways, they may themselves be targets of potential terrorism threats.

Our findings suggest rural LHD workers may have significantly higher odds of WTR across scenarios and WTR contexts than their urban counterparts (Table 2 and Table 3). Increased levels of social cohesion in rural communities may account for these increased WTR levels, although this warrants further study. Cohesive communities are characterized as sharing a common vision and sense of belonging, possessing an appreciation for the diversity of people's backgrounds, and developing strong and positive relationships among people with different backgrounds in neighborhoods, schools and workplaces [24].

According to Durkheim's theory of social solidarity, increased interdependence of individuals within a community may be related to increased levels of social cohesion [25]. This interdependence may be more pronounced in rural communities that act as independent microcosms of modern society, in which residents rely on one another for daily life essentials. An increased level of social cohesion may increase local public health workers' sense of belonging, duty, and responsibility to neighbors and coworkers, thus potentially explaining the presentation of increased WTR to an emergency situation among rural jurisdictions. Recent research has shown 29% more Florida healthcare workers reported WTR to a bioterrorism attack within their community than elsewhere in their state [26]. In light of these findings, and the lack of research on the effect of social cohesion on willingness to respond, the likelihood of the local public health workforce's emergency response in the context of social cohesion is seen as a potential area for future investigation.

Local public health agencies have a median staff size of 13 [22], which emphasizes the imperative that all workers are ready, willing, and able to respond to a public health emergency if needed [6]. The diminishing numbers of U.S. public health workers [14] reinforce the need to ensure that LHD employees are sufficiently committed to responding during an emergency. In six of the 12 WTR context/scenario combinations, the ORs that were statistically significant for WTR, comparing female to male respondents, ranged from 0.57 to 0.66. Since the public health workforce is comprised of 82.7% women [22], this finding has significant implications for local public health surge capacity. In addition, the public health workforce is aging. According to a recent Association of State and Tribal Health Officials (ASTHO) survey, 23% of the current workforce is eligible to retire in 2012 [27]. In eight of the 12 combinations, the significant ORs, comparing respondents at least 40 years of age to the younger respondents, ranged from 1.26 to 1.58. As more willing local public health responders approach retirement, it is now more important than ever to maximize the willingness of all available personnel to enhance the effectiveness of emergency response.

Given fiscal austerity challenges facing the public health infrastructure [28], efforts to increase funding for the LHD workforce may be extremely difficult. However, this study's findings can inform the augmentation of current LHD training programs and adaptation of agency policies. According to the EPPM, messages need to convey both threat and efficacy in order to elicit intended desirable protective behaviors [29]. Comparisons of WTR levels across EPPM categories consistently indicate that regardless of perceived threat, efficacy increased respondents' WTR (Table 4). The enhanced effect of efficacy, compared to threat, on WTR is consistent with findings from previous research [10,11]. Bandura has modeled behavior change and maintenance as a function of self-efficacy and response efficacy [30]. A review of the literature shows efficacy to have a strong association with the successful performance of myriad health behaviors, including cigarette smoking, weight control, exercise, and contraception use [31]. The importance of threat and efficacy in understanding emergency response behavior has been highlighted in recent research in Washington State healthcare workers. In this cohort, self-reported ability was higher than self-reported willingness to report to work for an influenza pandemic, but self-reported willingness was higher than self-reported ability for a severe earthquake event [32]. This distinction by scenario suggests that perceived threat and efficacy associated with each event has a distinct impact on behavior.

This study's finding of efficacy, as a crucial modifier of response willingness across scenarios, has important implications for designing novel preparedness curricula for LHD employees. Namely, our data suggest that such curricula could usefully employ a variety of efficacy-focused cognitive interventions to achieve the desired affective outcome of increased WTR.

Curricular interventions of this nature could include explicit emphasis on household preparedness-building activities for LHD employees with dependents. Personnel with dependents were shown to have lower odds of self-reported WTR for an emergency (range of ORs: 0.59 to 0.78) than those without this responsibility. To increase the level of WTR among this cohort, preparedness curricula can instruct LHD employees on the essentials of making family preparedness plans and kits, and potentially requiring employees to do so. While this may seem like an unfunded requirement, a curriculum that uses cognitive intervention for achieving affective outcomes could emphasize the benefit outweighing the cost of preparedness plans and kits, by highlighting their importance in optimizing the safety of dependents and enhancing employees' comfort level in reporting to work during an emergency.

LHD employees who perceived themselves as having important roles in their agency's emergency response efforts consistently had higher odds of self-reported WTR to all four surveyed scenarios regardless of severity. Workers who perceived themselves as likely to be asked by their agencies to report to duty also had higher odds of WTR if required. Accordingly, to boost willingness, preparedness curricula might aim to build employees' sense of response efficacy by highlighting how and why each of their roles is important, and emphasizing that each employee be considered a vital contributor to an agency's response. Curricula can enhance self-efficacy by instructing employees on the range of role-specific activities they may be expected to perform, and illustrating how their daily skill sets might transfer readily to this spectrum of response expectations. For example, finance support staff within LHDs would receive role-specific instruction and reassurance on how their everyday activities, (e.g., coordinating daily inventory and payroll) would apply to their public health emergency response efforts (e.g., managing emergency supply inventory and monitoring overtime payroll in a public health crisis). Staffing challenges facing LHDs [14] only heighten the importance of instilling a mindset of everyone being necessary in a team response, through efficacy-focused preparedness curricula.

Local public health agencies' policies also have the potential to enhance their workers' WTR. Geographic variations across clusters (Table S3) suggest a need for local WTR assessments as the basis for tailored curricular efforts to enhance response willingness rates in LHDs. Despite these variations, however, certain commonalities emerged with generalizable training implications. Across clusters, LHD workers consistently reported increased WTR if they perceived it to be a requirement (Table S3). LHDs may wish to reexamine their policies on employee requirements for responding to different emergencies in order to increase potential likelihood of response. The impact on WTR of policies requiring employees to work, including those imposed by unions, in oaths taken by sworn personnel, and by the health department, local government or state government, needs to be explored in future research.

Further, irrespective of geographic location or rural/urban status, WTR was consistently and markedly lowest for the radiological 'dirty' bomb scenario. This finding suggests a need for intensive and broad-scale efforts to enhance LHD employees' awareness of the role of public health in radiological emergency responses and augment their sense of efficacy in contributing to such responses. Clearly-articulated, LHD policies that directly address LHD employees' concerns about safety at work and at home may enhance their comfort level toward responding to radiological or other threats.

Certain limitations of this study must be acknowledged. First, results are based on survey data and may not be predictive of behavior in an actual, real-world response. Given the unpredictable variability of disasters and inability to control for various confounding factors, a prospective study was conducted to limit bias and yield the most methodologically sound results. Second, the recruitment of proximate LHDs to form the study's clusters was based on convenience and snowball sampling rather than random sampling, having the potential to limit generalizability. This research was not intended to provide representative results as from a nationwide survey, in which case the sampling design would have an impact on the results and associated confidence intervals. While a geographically and demographically diverse set of clusters was included in order to maximize external validity of our findings, conclusions may not be valid in locations not analyzed due to external factors, including past experiences with disaster scenarios or differences in local public health infrastructure. Third, while the four scenarios in the study reflect categories of high-consequence events, the surveyed scenarios do not reflect the entire all-hazards continuum. Fourth, the 2009 H1N1 influenza pandemic overlapped the survey window for all but one of the study's LHD clusters. Actual experience with this event may have influenced responses to this scenario category.

## Conclusions

"All disasters begin locally [33]" is a guiding maxim for preparedness and response. Planning for LHDs to figure centrally in emergency response requires efforts to increase the likelihood that this workforce will respond when asked to do so. Efficacy-focused curricular interventions with desired affective outcomes, coupled with amendments to agency policy based on scenario-specific and jurisdictional, demographic, and attitudinal differences, may have the potential to increase the response willingness of this vital cohort.

## Abbreviations

CI: Confidence interval; EPPM: Extended Parallel Process Model; JH ~ PHIRST: Johns Hopkins ~ Public Health Infrastructure Response Survey Tool; LHD: Local health department; OR: Odds ratio; WTR: Willingness to respond.

## Competing interests

The authors declare that they have no competing interests.

## Authors' contributions

DJB coordinated the study design, implementation, data interpretation, and full manuscript development. CBT led the analysis of the cross-sectional survey results in this study and drafted the Results section, and contributed to the Methods section. NAE was involved in drafting the Discussion and Conclusions sections, and contributed to interpretation of the analyzed survey data. NLS coordinated the survey implementation and contributed to drafting the Methods section. MKA contributed to the survey implementation, interpretation of data, and manuscript review and editing for intellectual content. JLF contributed to the survey implementation, interpretation of data, and manuscript review and editing for intellectual content. JMF contributed to the survey implementation, interpretation of data, and manuscript review and editing for intellectual content. RH contributed to the survey implementation, interpretation of data, and manuscript review and editing for intellectual content. MMK contributed to the survey implementation, interpretation of data, and manuscript review and editing for intellectual content. MM contributed to the survey implementation, interpretation of data, and manuscript review and editing for intellectual content. AME contributed to the survey implementation, interpretation of data, and manuscript review and editing for intellectual content. JS contributed to the survey implementation, interpretation of data, and manuscript review and editing for intellectual content. RDB contributed to survey design, interpretation of data, and manuscript review and editing for intellectual content. JML contributed to interpretation of survey data, along with manuscript review and editing for intellectual content. All authors read and approved the final manuscript.

## Pre-publication history

The pre-publication history for this paper can be accessed here:

http://www.biomedcentral.com/1471-2458/12/164/prepub

## Supplementary Material

Additional file 1 **Methodology1**. Click here for file
