# Supplementary material for: Determinants of emergency response willingness in the local public health workforce by jurisdictional and scenario patterns: a cross-sectional survey
Source: BMC Public Health. 2012 Mar 7;12:164. doi: 10.1186/1471-2458-12-164 (PMC3362768; doi:10.1186/1471-2458-12-164)
Supplement: Additional file 1 — Methodology1. [file 1471-2458-12-164-S1.PDF]

Methodology

Details of EPPM categorization:

Responses to four of the attitude/belief statements were used to assign a scenario-specific EPPM categorization for each survey participant.

Threat dimension statements for weather-related disaster:

A weather-related disaster is likely to occur in this region.

If it occurs, a weather-related disaster in this region is likely to have severe public health consequences.

Efficacy dimension statements for weather-related disaster, Self-efficacy and Response efficacy, respectively:

I would be able to perform my duties successfully in the event of a weather-related disaster.

If I perform my role successfully it will make a big difference in the success of the response to a weather-related disaster.

In order to have a scenario-specific EPPM categorization assigned, a participant had to have responses to all four of the above attitude/belief statements for that scenario. An initial score for each dimension (1-81) was then calculated as the cross-product of the original response scores (1-9) for its statements, with lower values indicating a stronger level of agreement with the dimension. This initial score was then dichotomized based on the median of the scenario-specific scores. Participants with scores below the median were categorized as High on the dimension, and participants with scores above the median were categorized as Low on the dimension. These dichotomized scores were then combined into one of the four EPPM categorizations:

Low Threat/Low Efficacy (LT/LE)

Low Threat/High Efficacy (LT/HE)

High Threat/Low Efficacy (HT/LE)

High Threat/High Efficacy (HT/HE)
